# Spontaneous Coronary Artery Dissection With Systemic Lupus Erythematosus

**DOI:** 10.31486/toj.22.0003

**Published:** 2022

**Authors:** Nourhan Chaaban, Shilpa Kshatriya

**Affiliations:** ^1^ Department of Internal Medicine, University of Kansas School of Medicine, Wichita, KS; ^2^ Heartland Cardiology, Wichita, KS

**Keywords:** *Lupus erythematosus–systemic*, *spontaneous coronary artery dissection*

## Abstract

**Background:** Spontaneous coronary artery dissection (SCAD) has been reported to be a rare cause of acute coronary syndrome and sudden cardiac death. The clinical presentation of SCAD varies from asymptomatic to sudden death. Pregnancy is associated with SCAD, and autoimmune diseases, especially systemic lupus erythematosus (SLE), may play an important role in SCAD etiology.

**Case Report:** A 37-year-old female with hypertension, SLE, a history of preeclampsia with 3 cesarean deliveries, and an active smoking habit presented to the emergency department with chest pain. On arrival, the patient was hypertensive with blood pressure of 152/122 mm Hg and a normal heart rate and respiratory rate. Given the patient's history of SLE and preeclampsia, antiphospholipid antibodies were tested. The anti-β_2_-glycoprotein 1 immunoglobulin G concentration was elevated at 30 U/mL, and lupus anticoagulant was positive. Electrocardiogram showed minimum ST elevation in lead V2. Initial troponin was 0.1 ng/mL, with a peak of 54.5 ng/mL after 6 hours. Aspirin 325 mg was administered, and the patient underwent urgent cardiac catheterization. Intravascular angiography showed evidence of intimal flap (mid left anterior descending artery) spontaneous dissection with subintimal hematoma. The angioplasty resulted in successful stent placement in the mid left anterior descending artery.

**Conclusion:** SCAD diagnosis is challenging and requires a high index of suspicion. This case shows the challenge of early diagnosis of SCAD and highlights its association with autoimmune diseases, specifically SLE. Early recognition of this pathology results in better outcomes.

## INTRODUCTION

Spontaneous coronary artery dissection (SCAD) has been defined as an intramural hematoma of the media of the vessel wall (false lumen) which flattens the true lumen, leading to blood flow obstruction and acute myocardial ischemia in the absence of trauma or iatrogenic causes.^1^ It has been reported to be a rare cause of acute coronary syndrome and sudden cardiac death. In fact, SCAD is the cause of acute coronary syndrome in 0.1% to 1.1% of cases.^2^ The clinical presentation of SCAD varies from asymptomatic to sudden death.^3^ The first report of SCAD was of the autopsy of a 42-year-old female in 1931.^4^ SCAD is an underdiagnosed disease that requires a high index of suspicion. We report a unique case to emphasize the benefit of early recognition of SCAD and to show the association of SCAD with systemic lupus erythematosus (SLE).

## CASE REPORT

A 37-year-old female with hypertension, SLE, a history of preeclampsia with 3 cesarean deliveries, and an active smoking habit presented to the emergency department (ED) with shortness of breath that started on the day of admission. The patient was having dinner and suddenly started experiencing diffuse chest pain that radiated to the bilateral upper extremities.

On arrival in the ED, the patient was hypertensive with blood pressure of 152/122 mm Hg. Heart rate and respiratory rate were normal. Electrocardiogram (ECG) showed minimum ST elevation in V2. Blood work was unremarkable with a white blood cell count of 5,000 cells/μL (reference range, 4,500-11,000 cells/μL), hemoglobin concentration of 14 g/dL (reference range, 12.1-15.1 g/dL), and platelet count of 170,000 cells/μL (reference range, 150,000-450,000 cells/μL). The patient's blood urea nitrogen concentration was 10 mg/dL (reference range, 6-24 mg/dL), and her creatinine concentration was 0.7 mg/dL (reference range, 0.6-1.1 mg/dL). Initial troponin level was 0.1 ng/mL (reference, <0.4 ng/mL). The patient was administered aspirin 325 mg and 2 sublingual nitroglycerin tablets of 0.3 mg each. The patient experienced some relief from her chest pain and was admitted to the intensive care cardiology unit. Troponin level was trended, and it peaked after 6 hours at 54.5 ng/mL.

Given the patient's history of SLE and preeclampsia, antiphospholipid antibodies were tested. The anti-β_2_-glycoprotein 1 immunoglobulin G (IgG) concentration was elevated at 30 U/mL (reference range, 0-20 U/mL), and lupus anticoagulant was positive. Antinuclear antibody and antineutrophil cytoplasmic antibody were absent. Inflammatory markers including C-reactive protein concentration and erythrocyte segmentation rate were not elevated.

After 10 hours, the patient was started on a heparin drip and taken to the cardiac catheterization laboratory for acute coronary disease management. A 6 French guiding catheter inserted in the right groin showed total occlusion of the mid left anterior descending coronary artery. A run-through wire was advanced to the first diagonal branch, and a second wire was advanced to the distal left anterior descending artery. Coronary angiogram showed evidence of intimal flap (mid left anterior descending artery) spontaneous dissection with subintimal hematoma (Figure). The mid left anterior descending artery lesion was long with moderate stenosis. Successful percutaneous coronary intervention and stent placement with intravascular ultrasound guidance were performed, and a 4.0 × 34 mm drug-eluting stent was deployed in the mid left anterior descending artery and post-dilated with a 4.5 mm noncompliant balloon. A percutaneous closure device (Angio-Seal VIP, Terumo Interventional Systems) was successfully placed in the right femoral artery.

**Figure.**
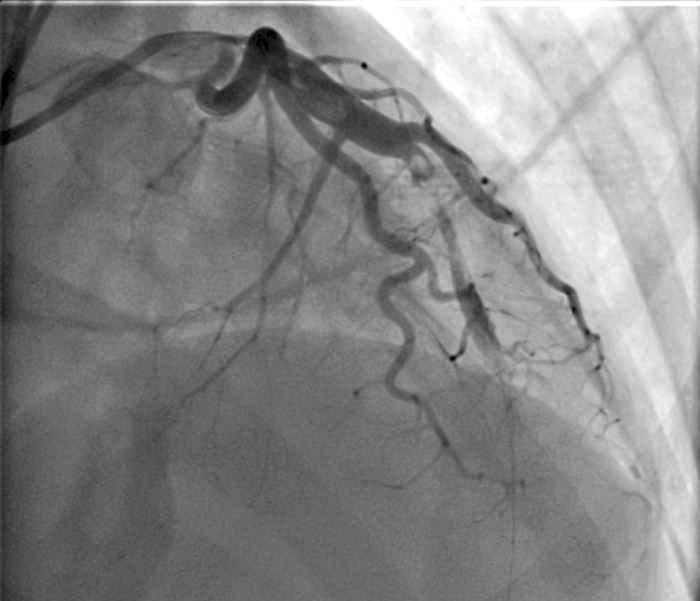
Cardiac catheterization shows total occlusion of the mid left anterior descending coronary artery with intimal flap spontaneous dissection with subintimal hematoma.

The angioplasty resulted in successful stent placement in the mid left anterior descending artery. The length of the lesion was approximately 30 mm, and it appeared to be secondary to spontaneous intimal dissection. No evidence of atherosclerosis was noted.

The patient was started on aspirin 81 mg and clopidogrel 75 mg daily, atorvastatin 40 mg daily in the evening, metoprolol 25 mg daily, and lisinopril 20 mg daily. Echocardiogram 2 days after heart catheterization revealed a low ejection fraction of 20% to 25%. The patient was given heart failure education including instructions for a low sodium diet, medication compliance, and moderate exercise activity.

The patient was discharged after 7 days of hospitalization on warfarin 5 mg daily with a follow-up scheduled with the rheumatology team. She was seen in the cardiology clinic 2 weeks after discharge. The patient was doing well and reported no concerns. Echocardiogram 3 months later showed an improved ejection fraction of 35% to 40%.

After 12 months, per rheumatology request, workup for antiphospholipid antibodies, including the lupus anticoagulant, was repeated, with negative findings. Anti-β2-glycoprotein 1 IgG and lupus anticoagulant were within normal range. The decision of the rheumatology team was to maintain the patient on warfarin therapy with international normalized ratio monitoring every 2 to 4 weeks.

## DISCUSSION

The underlying mechanism of nonatherosclerotic SCAD remains uncertain. Two potential mechanisms for the initiation of arterial wall separation have been proposed. The first is the intimal tear hypothesis, in which a primary disruption in the intimal luminal interface creates an entry point for intramural hematoma (IMH) accumulation inside the false lumen, leading to separation of the arterial wall.^5^ The second is the medial hemorrhage hypothesis, in which a hemorrhage into the arterial wall is the primary mechanism, perhaps due to spontaneous rupture from the increased density of the vasa vasorum.^6^ Both result in the creation of a false lumen filled with intramural hematoma.

Pregnancy is associated with SCAD. A 2017 analysis of a US administrative database found a prevalence of 1.81 SCAD events per 100,000 pregnancies during pregnancy or in the 6-week postpartum period.^7^ The increased risk of SCAD during pregnancy had been attributed to the hormonal, hemodynamic, and hematologic changes that accompany pregnancy.^8^ Additionally, one case report shows an association between preeclampsia and SCAD.^9^ The pathogenesis behind this association remains unknown.

Moreover, autoimmune diseases, especially SLE, may play an important role in SCAD etiology. Patients with SLE have a 50-fold increase in the prevalence of coronary artery disease compared to an age-matched control group.^10^ Few published case reports have highlighted this association.^10-13^ One of the proposed mechanisms behind this association is similar to the inflammatory pathophysiology seen with vasculitis. Data from anatomopathologic studies showed the presence of the periarterial eosinophilic infiltrate in patients with SCAD and confirmed its absence in patients with iatrogenic or traumatic dissections.^14^ Interestingly, a case has also been published about a patient with SCAD and positive antiphospholipid antibodies.^11^

Ullah et al conducted a systematic review of 10 articles about SCAD associated with rheumatologic conditions and found that many cases were associated with systemic lupus, most patients presented with non-ST elevation myocardial infarction involving the left main coronary artery, and the majority were managed with stenting.^15^ Mortality was <20%.

However, the data on the association between rheumatologic conditions and SCAD are scarce; no randomized trials exist on this topic, so only assumptions can be made that patients with rheumatologic conditions have a high risk for SCAD. The role of inflammatory markers and their impact on coronary vessel walls is a hypothesis that needs a large-scale study. Patients with atypical chest pain and young females with myocardial infarction and subtle ECG changes in the presence of rheumatologic conditions should be investigated for SCAD. In our case, the patient had risk factors of preeclampsia, tested positive for antiphospholipid antibodies, and presented with typical acute coronary syndrome symptoms, chest pain radiating to bilateral upper extremities.

The gold standard for the diagnosis of SCAD is coronary angiography. SCAD exhibits 3 main patterns: the type 1 angiographic SCAD appearance, although it is not the most common pattern, can be demonstrated well on angiography with multiple radiolucent and contrast dye stains in the artery wall; the type 2 angiographic SCAD appearance is the most common, with long diffuse stenosis of varying severity, commonly in the mid to distal segments; and the type 3 angiographic appearance is challenging to diagnose because it mimics atherosclerosis.^16^ Techniques such as intravascular ultrasound guidance and optical coherence tomography are alternatives to coronary angiography for the visualization and identification of SCAD.

No specific guidelines have been developed for the management of SCAD. Treatment options for SCAD include medical therapy, percutaneous coronary intervention, or coronary artery bypass graft surgery.^17^ Medical management of SCAD is similar to the management of acute coronary syndrome management, including antithrombotic therapy with heparin or low molecular weight heparin, aspirin, clopidogrel, and anti-ischemic therapy.

## CONCLUSION

Making a SCAD diagnosis is challenging and requires a high index of suspicion. Our patient with SLE presented with chest pain and was diagnosed with SCAD. This case highlights the association of SCAD with autoimmune diseases, especially SLE, with positive antiphospholipid antibodies. Clinicians need to consider patients with high-risk features, in our case lupus, for SCAD diagnosis.

## Notes

*This article meets the Accreditation Council for Graduate Medical Education and the American Board of Medical Specialties Maintenance of Certification competencies for Patient Care and Medical Knowledge.*
